# Gremlin-1 is a key regulator of the invasive cell phenotype in mesothelioma

**DOI:** 10.18632/oncotarget.21550

**Published:** 2017-10-06

**Authors:** Miao Yin, Mira Tissari, Jenni Tamminen, Irene Ylivinkka, Mikko Rönty, Pernilla von Nandelstadh, Kaisa Lehti, Marko Hyytiäinen, Marjukka Myllärniemi, Katri Koli

**Affiliations:** ^1^ Research Programs Unit, Translational Cancer Biology, University of Helsinki, Helsinki, Finland; ^2^ Transplantation Laboratory, Haartman Institute, University of Helsinki, Helsinki, Finland; ^3^ Department of Pathology, University of Helsinki and Fimlab Laboratories, Pathology, Tampere, Finland; ^4^ Research Programs Unit, Genome Scale Biology, University of Helsinki, Helsinki, Finland; ^5^ K. Albin Johansson Foundation, Finnish Cancer Institute, Helsinki, Finland; ^6^ Department of Microbiology, Tumor and Cell Biology, Karolinska Institute, Solna, Sweden; ^7^ Department of Pulmonary Medicine, University of Helsinki and Helsinki University Hospital, Heart and Lung Center, Helsinki, Finland

**Keywords:** gremlin, mesothelioma, invasion

## Abstract

Malignant mesothelioma originates from mesothelial cells and is a cancer type that aggressively invades into the surrounding tissue, has poor prognosis and no effective treatment. Gremlin-1 is a cysteine knot protein that functions by inhibiting BMP-pathway activity during development. BMP-independent functions have also been described for gremlin-1. We have previously shown high gremlin-1 expression in mesothelioma tumor tissue. Here, we investigated the functions of gremlin-1 in mesothelioma cell migration and invasive growth. Gremlin-1 promoted mesothelioma cell sprouting and invasion into three dimensional collagen and Matrigel matrices. The expression level of gremlin-1 was linked to changes in the expression of SNAI2, integrins, matrix metalloproteinases (MMP) and TGF-β family signaling - all previously associated with a mesenchymal invasive phenotype. Small molecule inhibitors of MMPs completely blocked mesothelioma cell invasive growth. In addition, inhibitors of TGF-β receptors significantly reduced invasive growth. This was associated with reduced expression of MMP2 but not SNAI2, indicating that gremlin-1 has both TGF-β pathway dependent and independent mechanisms of action. Results of *in vivo* mesothelioma xenograft experiments indicated that gremlin-1 overexpressing tumors were more vascular and had a tendency to send metastases. This suggests that by inducing a mesenchymal invasive cell phenotype together with enhanced tumor vascularization, gremlin-1 drives mesothelioma invasion and metastasis. These data identify gremlin-1 as a potential therapeutic target in mesothelioma.

## INTRODUCTION

Malignant mesothelioma is an aggressive cancer that develops from the mesothelial cells of serosal membranes [1]. Most often mesothelioma originates from the pleural lining of the lung. Occupational or incidental exposure to asbestos fibers is a known causative factor in mesothelioma and the latency period from exposure to tumor incidence can be long [2]. Mesothelioma prognosis is poor, survival time being usually only 10–14 months after diagnosis [3]. There are three histological subtypes of mesothelioma. Epithelioid mesothelioma is the most common type with prominent papillotubular structures seen in histological micrographs. Sarcomatoid mesothelioma is characterized by spindled cells mimicking fibrosarcoma. Biphasic mesothelioma has areas of both epithelioid and sarcomatoid histology. Mesothelioma shows aggressive local growth and invasion into the chest wall, diaphragm or contralateral lymph nodes [1]. Distant metastases are occationally detected. Mesothelioma is highly resistant to conventional cancer therapies.

Invasive processes are regulated by the tumor microenvironment, where the extracellular matrix molecules and secreted growth factors contribute to the transition of tumor cells towards a migratory and invasive phenotype. It is noteworthy that tumor cell invasion and metastases may be an unrelated process to tumor cell proliferation and occur already at early stages of tumor development [4]. Therefore, it is essential to identify molecules that potentiate mesothelioma invasion and dissemination. Gremlin-1 is a member of the DAN family of BMP antagonists, which inhibits mainly the activity of BMP-2 and -4 by direct binding to the growth factor [5]. Gremlin-1 plays essential roles especially during kidney, lung and bone development [6]. It has been identified as a niche factor contributing to stem cell proliferation by blocking BMP signaling [7]. BMP-independent functions, such as modulation of VEGFR2-dependent induction of angiogenesis and inhibition of macrophage migration inhibitory factor (MIF), have also been described for gremlin-1 [8, 9]. Recently, gremlin-1 expression has been associated with tumorigenesis. Gremlin-1 was found to be expressed by cancer-associated stromal cells in the tumor tissues and this way thought to contribute to a microenvironment regulated tumor growth and invasion [10]. We and others have found gremlin-1 to be expressed by the tumor cells [11]. Our previous studies suggest that mesothelioma cells secrete high amounts of gremlin-1, which is targeted into fibrillin-2 rich microfibrils in the mesothelioma tumor tissue [12].

To better understand the functions of gremlin-1 in mesothelioma we assessed mesothelioma cell migration and invasion *in vitro* and *in vivo*. Mesothelioma invasion into 3D collagen matrix was dependent on gremlin-1 expression. Gremlin-1 associated with the expression of the EMT transcription factor *SNAI2*, modulation of proteolytic enzyme expression and increase in TGF-β pathway activity. Data from *in vivo* tumor xenograft experiments indicated a vascular phenotype and a tendency to send metastases in gremlin-1 expressing tumors. These results indicate that gremlin-1 drives invasion and dissemination in mesothelioma.

## RESULTS

### Gremlin-1 drives invasive growth of mesothelioma cells in 3D culture

**Figure 1:**
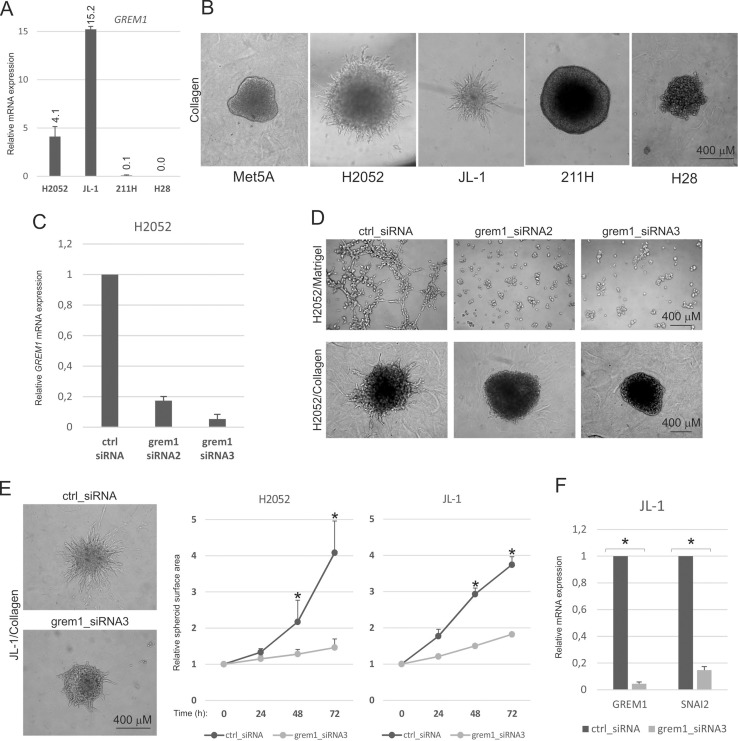
Gremlin-1 regulates 3D invasion of mesothelioma cell lines (**A**) H2052, JL-1 and 211H mesothelioma cells were analyzed for *GREM1* expression by quantitative RT-PCR. The level was normalized to the expression levels of TATA-binding protein and is expressed relative to the expression level in Met5A (immortalized, non-tumorigenic mesothelial cells), which was set to 1. The error bars represent SD (*n* = 3). (**B**) Invasive growth of Met5A control cells and mesothelioma cell lines was analyzed in three-dimensional (3D) collagen 1 matrix. Cells were embedded into the matrix as spheroids and followed up to 72 hours. (**C**) *GREM1* expression was analyzed in control siRNA (ctrl_siRNA) and gremlin-1 siRNA (grem1_siRNA) transfected cells 72 hours after transfection. The results are expressed relative to the expression level in ctrl_siRNA transfected cells, which was set to 1. The error bars represent SD (*n* = 3). (**D**) Invasive growth of gremlin-1 silenced H2052 cells was analyzed in 3D Matrigel or collagen 1 matrix. (**E**) Invasive growth of gremlin-1 silenced JL-1 cells was analyzed in 3D collagen 1 matrix. Images were taken at 72 hours. Graphs show quantification as relative spheroid surface area. The error bars represent SD (*n* = 3). ^*^p < 0.05. (**F**) Relative expression of *GREM1* and *SNAI2* in control siRNA (ctrl_siRNA) and gremlin-1 siRNA (grem1_siRNA) transfected cells 72 hours after transfection. The error bars represent SD (*n* = 3). ^*^p < 0.05.

Since mesothelioma tumors are highly invasive locally, we analyzed whether gremlin-1 regulates invasive growth of mesothelioma cells. H2052 and JL-1 mesothelioma cell lines, which express higher mRNA levels of gremlin-1 than non-tumorigenic and non-invasive Met5A control cells (Figure 1A), showed invasive sprouting when tumor cell spheroids were imbedded into 3D collagen matrix (Figure 1B). H28 and 211H cells with undetectable or low gremlin-1 expression were not invasive under similar conditions. Gremlin-1 expression was silenced in H2052 cells using two different siRNAs. Both siRNAs reduced gremlin-1 mRNA expression significantly, siRNA3 being more effective with 95% reduction of expression (Figure 1C). Control siRNA treated cells embedded into 3D Matrigel were able to form irregular shaped colonies and invade and sprout through the surrounding matrix (Figure 1D). Gremlin-1 silencing efficiently inhibited both Matrigel and collagen invasion of H2052 cells (Figure 1D, 1E). Similar reduction in collagen invasion was noted in gremlin-1 silenced JL-1 cells, which were followed up to 72 h after embedding of cell spheroids into 3D collagen (Figure 1E). In addition, gremlin-1 silencing resulted in downregulation of the expression of the EMT transcription factor *SNAI2* (Figure 1F), similar to what we have reported previously in H2052 cells [12].

**Figure 2:**
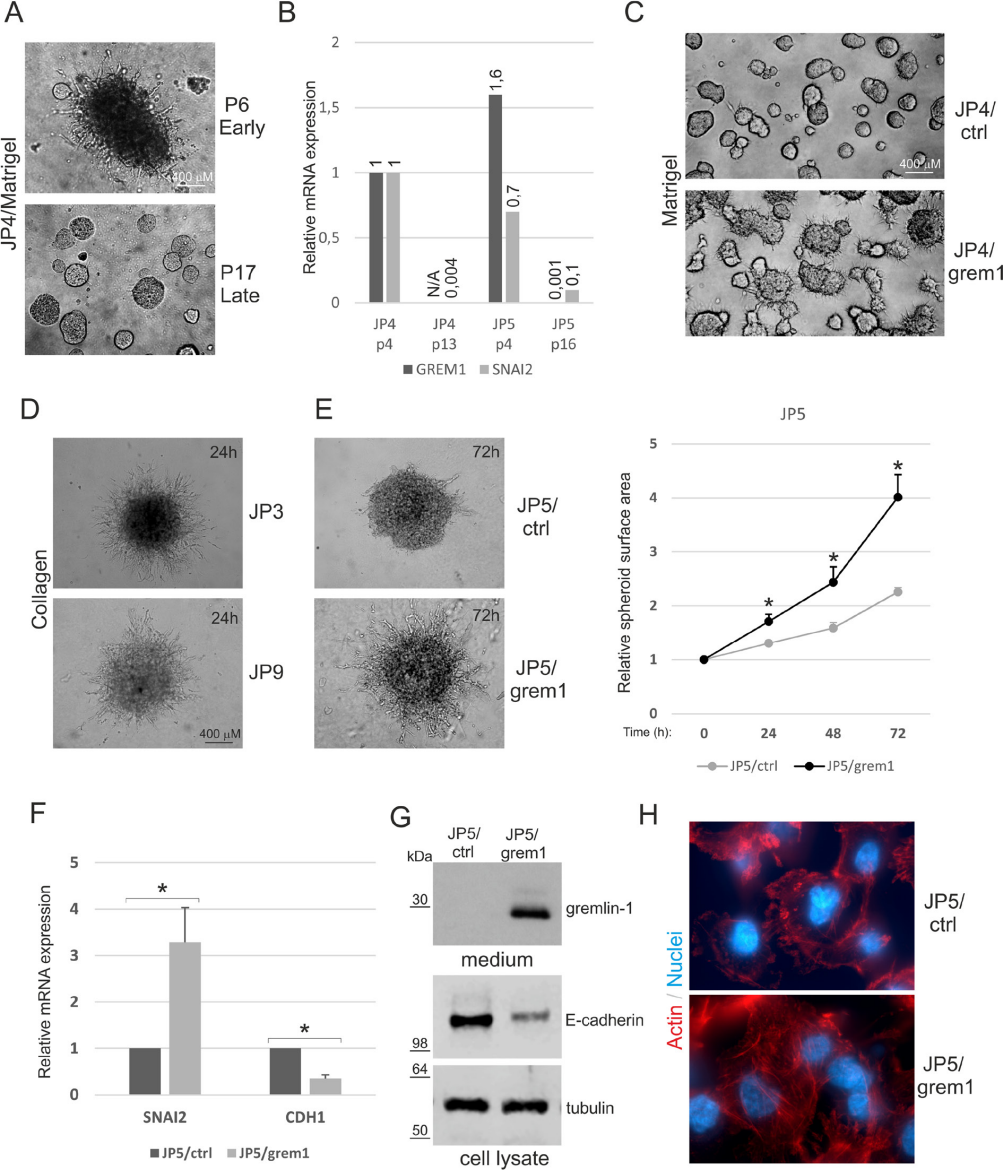
Rescue of 3D invasive growth by gremlin-1 overexpression (**A**) Early and late passage JP4 mesothelioma cells were embedded into 3D Matrigel and followed for 72 hours. (**B**) Expression of *GREM1* and *SNAI2* in early and late passage JP4 and JP5 mesothelioma cells. The results are expressed relative to the expression level in early passage JP4 cells, which was set to 1. A representative experiment is shown. (**C**) Late passage JP4 mesothelioma cells transduced to express gremlin-1 (JP4/grem1) or control cells (JP4/ctrl) were embedded into 3D Matrigel and followed for 72 hours. (**D**–**E**) Early passage JP3 and JP9 primary mesothelioma cells and JP5 mesothelioma cells transduced to express gremlin-1 (JP5/grem1) or control cells (JP5/ctrl) were embedded into 3D collagen 1. Images were taken at 24 or 72 hours. Graphs shows quantification as relative spheroid surface area. The error bars represent SD (*n* = 3). ^*^p < 0.05. (**F**) Expression of *SNAI2* and *CDH1/E-cadherin* in JP5/ctrl and JP5/grem1 cells. The results are expressed relative to the expression level in JP5/ctrl cells, which was set to 1. The error bars represent SD (*n* = 3). ^*^p < 0.05. (**G**) Cell conditioned media (normalized according to cell number) or cell lysates were analyzed by Western blotting using antibodies specific to gremlin-1, E-cadherin or β-tubulin (loading control). The molecular weight markers (kDa) are shown on the left. (**H**) JP5/ctrl and JP5/grem1 cells were stained for F-actin (red) and analyzed using immunofluorescence microscopy. Cell nuclei were counterstained with DAPI (blue).

Primary mesothelioma cells isolated from pleural effusions of mesothelioma patients express high mRNA levels of gremlin-1 [12]. We noticed that primary cells initially grew slowly, but when passaged more than 10 times the growth was gradually increased (not shown). JP4 early passage cells, but not late passage cells, were able to sprout and invade into 3D Matrigel (Figure 2A). This change in 3D phenotype was associated with downregulation of *GREM1* mRNA expression (Figure 2B). In late passage JP4 and JP5 cells there was a concomitant decrease in the expression level of *SNAI2*, which is consistent with our previous results linking gremlin-1 to a mesenchymal mesothelioma phenotype. When gremlin-1 was re-expressed in JP4 and JP5 late passage cells using lentiviral expression vectors, there was a gain of invasive growth and sprouting into 3D Matrigel (Figure 2C, Supplementary Figure 1).

Early passage primary mesothelioma cells showed robust invasive sprouting into 3D collagen matrix already at 24 hour time point (Figure 2D). Furthermore, late passage JP5 cells transduced to re-express gremlin-1 (JP5/grem1) were also invasive in 3D collagen, while JP5 control cells (JP5/ctrl) were not (Figure 2E). The results indicate that the ability of mesothelioma cells to invade into Matrigel or collagen matrices is linked to gremlin-1 expression. Gremlin-1 re-expression was associated with increased levels of *SNAI2* and decreased levels of *CDH1* mRNA expression (Figure 2F). E-cadherin protein levels were 5-fold higher in JP5/ctrl cells, indicating a significant decrease by gremlin-1 expression (Figure 2G). In addition, the formation of actin stress fibers was observed in JP5/grem1 cells (Figure 2H). This suggests that gremlin-1 regulates the invasive growth of mesothelioma cells by driving a mesenchymal phenotype.

### Mesothelioma cell adhesion and migration is regulated by gremlin-1

**Figure 3:**
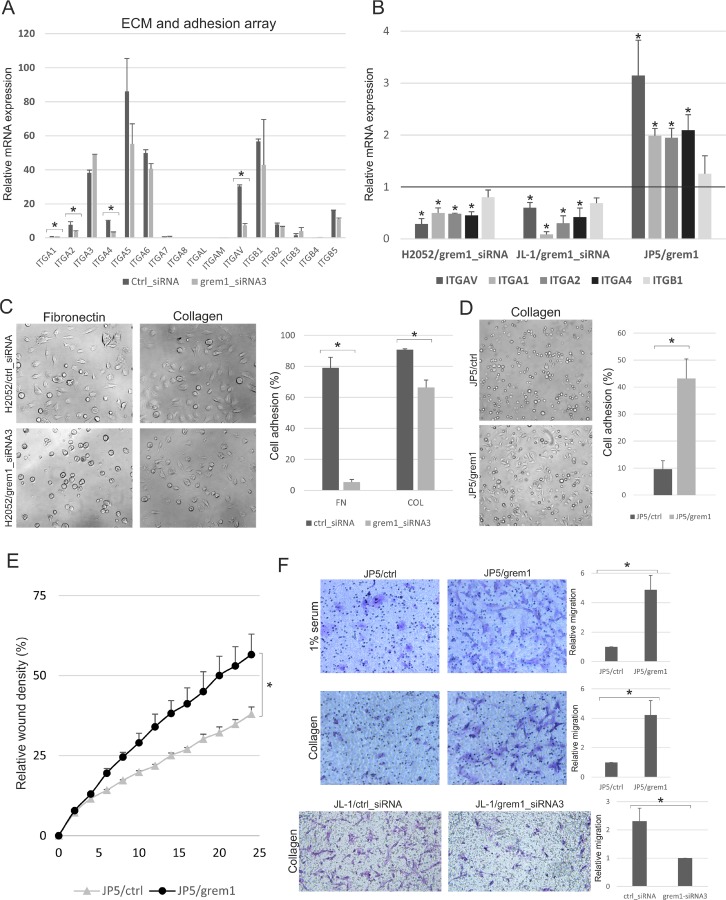
Mesothelioma cell adhesion and migration is regulated by gremlin-1 (**A**) Extracellular matrix and adhesion molecule PCR array was used to analyze gene expression in control siRNA (ctrl_siRNA) or gremlin-1 siRNA (grem1_siRNA3) transfected H2052 cells 3 days after transfection. The results are expressed relative to the expression level of *ITGA1* in H2052 control siRNA transfected cells, which was set to 1. The error bars represent SD (*n* = 2). (**B**) Expression of integrin genes in gremlin-1 silenced (grem1_siRNA) H2052 or JL-1 cells and in JP5/grem1 cells. The results are expressed relative to each control (control siRNA transfected or JP5/ctrl), which was set to 1. The error bars represent SD (*n* = 3). ^*^p < 0.05. (**C**) Control siRNA (ctrl_siRNA) or gremlin-1 siRNA (grem1_siRNA3) transfected H2052 cells were allowed to adhere to fibronectin or collagen 1 coated wells for 1 hour. (**D**) JP5/ctrl or JP5/grem1 cells were allowed to adhere to collagen 1 for 30 min. Quantification of cells with a spread/elongated morphology is shown. The error bars represent SD (*n* = 3). ^*^p < 0.05. (**E**) Cell migration was analyzed using a scratch-wound assay and monitored by 24-h live cell imaging. The results are presented as relative wound density. The error bars represent SD (*n* = 2). (**F**) Migration (1% serum as chemoattractant) or invasive migration (collagen 1 coated Transwell inserts) of JP5/ctrl, JP5/grem1 and JL-1 cells transfected with control or gremlin-1 siRNA is shown. Migrated cells were fixed, stained and imaged 16 hours after seeding. Graphs represent quantification of relative migration. The error bars represent SD (*n* = 3). ^*^p < 0.05.

We observed that silencing or overexpression of gremlin-1 in mesothelioma cells affected the adhesion of cells in 2D cultures. Therefore, we used a commercial PCR array to analyze the mRNA expression levels of extracellular matrix and adhesion molecules in gremlin-1 silenced H2052 cells (see Methods). Gremlin-1 silencing reduced the expression of several mesenchymal matrix proteins including *FN1*, *SPARC* and *TNC* (Supplementary Figure 2). In addition, the expression of integrin subunits was downregulated. *ITGAV*, collagen integrin alpha subunits *ITGA1* and *ITGA2* as well as *ITGA4* were all significantly downregulated (Figure 3A). More detailed quantitative RT-PCR analyses confirmed that these integrin subunits were downregulated in gremlin-1 silenced JL-1 and H2052 cells and upregulated in JP5/grem1 cells (Figure 3B). The expression of the integrin beta subunit *ITGB1* was not significantly altered.

Integrins play an essential role in cancer cell attachment and migration. Integrin αv subunit forms fibronectin binding heterodimers. Therefore cell attachment assays using fibronectin or collagen 1 were performed next. We observed significantly reduced binding of H2052 cells to fibronectin when gremlin-1 was silenced (Figure 3C). However, integrin αv blocking antibody or the integrin inhibitor cilengitide (20 μM) were not able to reduce H2052 cell invasion into 3D collagen (not shown). H2052 cell adhesion to collagen 1 was also significantly reduced by gremlin-1 silencing, although to a lesser extent (Figure 3C). JP5/grem1 cells showed increased adhesion to collagen 1 as well as increased migration in a wound healing assay (Figure 3D–3E). In addition, JP5 cells/grem1 cells showed increased migration/invasion in a Transwell assay with 1% serum as a chemoattractant or using collagen 1 coating of the inserts (Figure 3F). Gremlin-1 silencing in JL-1 cells decreased migration/invasion through collagen 1 coated inserts. The results suggest that modulation of integrin expression and cell adhesion is linked to gremlin-1 induced change in mesothelioma cell phenotype.

### MMP activity is crucial for gremlin-1 induced 3D invasion

**Figure 4:**
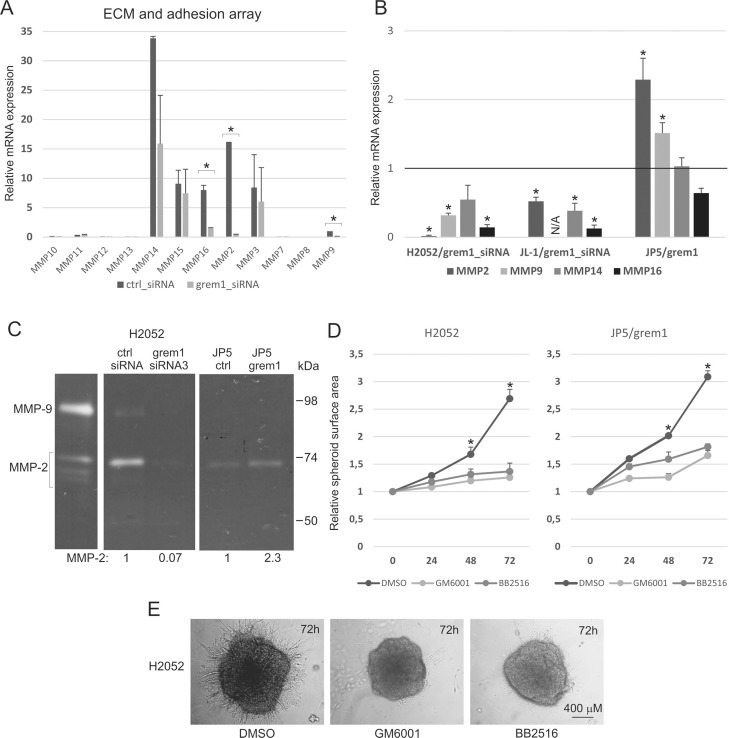
MMP activity is crucial for gremlin-1 induced 3D collagen invasion (**A**) Extracellular matrix and adhesion molecule PCR array was used to analyze gene expression in control siRNA (ctrl_siRNA) or gremlin-1 siRNA (grem1_siRNA3) transfected H2052 cells 3 days after transfection. The results are expressed relative to the expression level of *MMP9* in H2052 control siRNA transfected cells, which was set to 1. The error bars represent SD (*n* = 2). (**B**) Expression of MMP genes in gremlin-1 silenced (grem1_siRNA) H2052 or JL-1 cells and in JP5/grem1 cells. The results are expressed relative to each control (control siRNA transfected or JP5/ctrl), which was set to 1. The error bars represent SD (*n* = 3). ^*^p < 0.05. (**C**) Cell conditioned media (normalized to cell number) were analyzed by gelatin zymography. MMP-9 and MMP-2 (inactive, intermediate and active forms) in control sample (PMA treated HT1080 lysate) are shown on the left, molecular weight markers (kDa) on the right. Quantification of MMP-2 level is indicated from a representative experiment. (**D**) Invasive growth of H2052 and JP5/grem1 cells was analyzed in 3D collagen 1 matrix in the presence of DMSO or MMP inhibitors (GM6001 or BB2516 at 10 μM concentration). Graphs show quantification as relative spheroid surface area. The error bars represent SD (*n* = 3). ^*^p < 0.05. (**E**) Images from H2052 cells treated with DMSO or MMP inhibitors were taken at 72 hours.

The PCR array revealed also downregulation of the expression of several matrix metalloproteinases in gremlin-1 silenced H2052 cells (Figure 4A). Especially *MMP2*, *MMP9*, and *MMP16* expressions were significantly downregulated, *MMP14* also showing a trend towards reduced expression. Relative expression of different MMPs varied to some extent in the cell lines; however, *MMP14* (MT1-MMP) was the most abundantly expressed MMP while *MMP9* levels were low (Supplementary Figure 3). Quantitative RT-PCR analyses confirmed that several MMPs were downregulated in gremlin-1 silenced H2052 and JL-1 cells. However, although *MMP2* and *MMP9* expressions were upregulated in JP5/grem1 cells, the expression of membrane type MMPs (MMP14 and MMP16) was not increased (Figure 4B). Gelatin zymography assay indicated that MMP-2 and MMP-9 proteins were downregulated in gremlin-1 silenced H2052 cells and that MMP-2 protein levels were upregulated in JP5/grem1 cell conditioned medium (Figure 4C). The invasive growth of H2052 and JP5/grem1 cells into 3D collagen matrix was blocked by broad spectrum MMP inhibitors (GM6001 and BB2516) confirming the role of MMPs in gremlin-1 induced invasive growth (Figure 4D–4E).

### Activin-A and TGF-β expression is linked to gremlin-1 induced invasive growth

**Figure 5:**
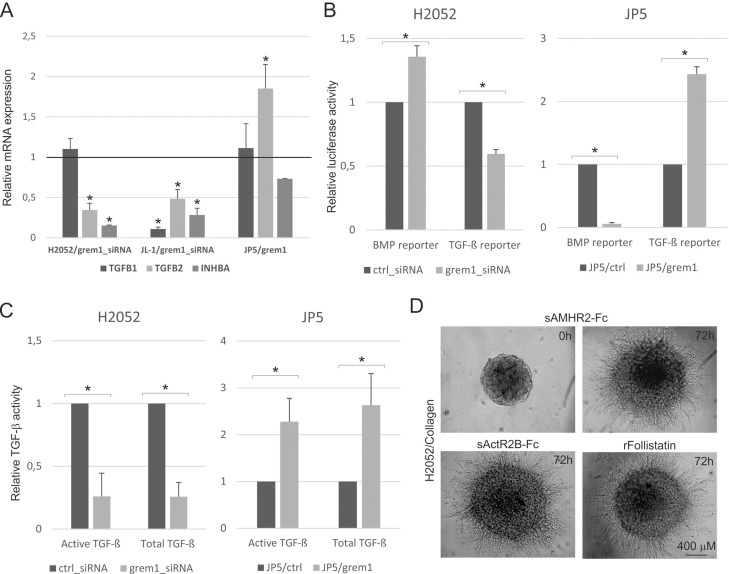
TGF-β pathway activity contributes to gremlin-1 induced invasive growth (**A**) Expression of TGF-β (TGFB1 and TGFB2) and activin-A (INHBA subunit) genes in gremlin-1 silenced (grem1_siRNA) H2052 or JL-1 cells and in JP5/grem1 cells. The results are expressed relative to each control (control siRNA transfected or JP5/ctrl), which was set to 1. The error bars represent SD (*n* = 3). ^*^p < 0.05. (**B**) BMP [(Bre)_2_-luc] and TGF-β [(CAGA)_12_-luc] pathway activities were analyzed using promoter reporter assays. Promoter activities in gremlin-1 siRNA (grem1_siRNA3) transfected H2052 cells or JP5/grem1 cells are shown relative to the levels in control siRNA (ctrl_siRNA) transfected or JP5/ctrl cells, respectively. The error bars represent SD (*n* = 3). ^*^p < 0.05. (**C**) Secreted levels of active and total TGF-β were analyzed using TGF-β responsive reporter cells. The amount of cell conditioned media was normalized according to cell number. TGF-β levels in gremlin-1 silenced (grem1_siRNA) H2052 cells and JP5/grem1 cells are expressed relative to each control (control siRNA transfected or JP5/ctrl), which was set to 1. The error bars represent SD (*n* = 3). ^*^p < 0.05. (**D**) Invasive growth of H2052 cells treated with a soluble inhibitor of activin signaling (sActR2B-Fc), recombinant follistatin or control inhibitor (sAMHR2-Fc, 0 h and 72 h time points shown) was analyzed in 3D collagen 1 matrix. Cells were followed up to 72 hours.

We and others have previously linked activin and TGF-β isoform expression to mesothelioma [13]. Reciprocal regulation of gremlin-1 and TGF-β pathway activity has also been reported [14, 15]. In addition, many TGF-β pathway genes were found to be downregulated in gremlin-1 silenced cells. Therefore, we analyzed the mRNA expression levels of *INHBA* (activin-A subunit), *TGFB1* and *TGFB2* in mesothelioma cells. Gremlin-1 silencing significantly reduced *TGFB2* and *INHBA* expression but did not alter *TGFB1* expression in H2052 cells (Figure 5A). In JL-1 cells gremlin-1 silencing reduced the expression of all isoforms, *TGFB1* being the most downregulated gene. JP5/grem1 cells, on the other hand, expressed more *TGFB2*, suggesting a link between gremlin-1 and activin/TGF-β isoform expression.

The effects of gremlin-1 silencing on TGF-β and BMP-pathway activities were studied using promoter luciferase constructs (see Methods). BMP-pathway activity was increased and TGF-β-pathway activity decreased in H2052 cells upon gremlin-1 knockdown (Figure 5B). In JP5 cells gremlin-1 overexpression led to a dramatic decrease in BMP-pathway activity as expected. TGF-β-pathway activity was increased. Secretion of active and latent forms of TGF-β into the cell culture medium were analyzed using reporter cells (see Methods). Silencing gremlin-1 in H2052 cells reduced and overexpression of gremlin-1 in JP5 cells increased the secretion of both active and total TGF-β (Figure 5C). This is in agreement with the promoter studies and is evidence of altered TGF-β/BMP signaling balance in mesothelioma cells.

**Figure 6:**
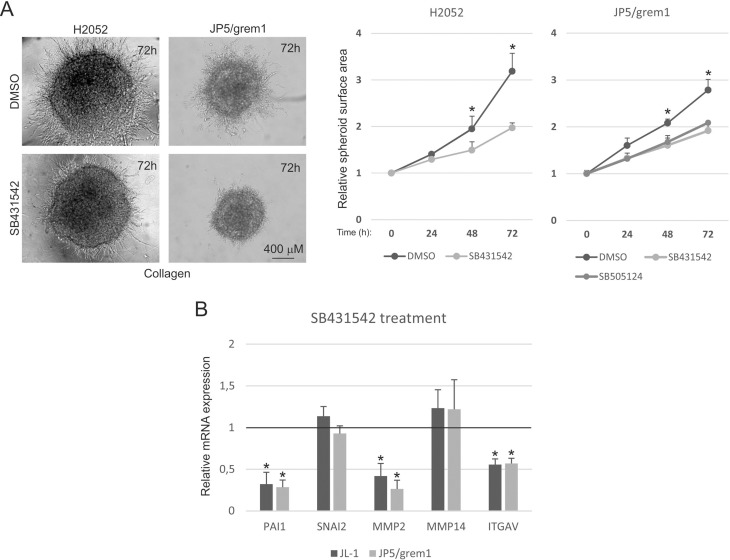
TGF-β pathway receptor inhibitors reduce gremlin-1 induced 3D collagen invasion (**A**) Invasive growth of H2052 and JP5/grem1 cells was analyzed in 3D collagen 1 matrix in the presence of DMSO or TGF-β/activin receptor inhibitors (SB431542 or SB505124 at 10 μM concentration). Graphs show quantification as relative spheroid surface area. The error bars represent SD (*n* = 3). ^*^p < 0.05. (**B**) Expression of *PAI1*, *SNAI2*, *MMP2,* *MMP14* and *ITGAV* in SB431542 treated mesothelioma cells (JL-1 and JP5/grem1). The results are expressed relative to each control (DMSO treated cells), which was set to 1. The error bars represent SD (*n* = 3). ^*^p < 0.05.

We have previously shown that inhibition of activin activity using a soluble activin receptor 2B (sActR2B-Fc) reduces mesothelioma cell migration and invasive growth in 3D Matrigel matrix [16]. However, blocking activin activity with sActR2B-Fc or recombinant follistatin were alone not able to inhibit 3D collagen invasion of H2052 cells (Figure 5D). Inhibition of activin/TGF-β pathway receptors (ALK4, ALK5 and ALK-7) by small molecule inhibitors (SB431542 and SB505124) reduced 3D collagen invasion of H2052 and JP5/grem1 cells (Figure 6A) suggesting a role for activin/TGF-β pathways in gremlin-1 induced invasive growth. In agreement, treatment of JL-1 and JP5/grem1 cells with SB431542 for 2 days reduced the mRNA expression of TGF-β target gene *PAI1* as well as *MMP2* and *ITGAV*. However, *SNAI2* and *MMP14* expressions were not reduced indicating also TGF-β pathway independent functions for gremlin-1.

### Inhibition of BMP receptor pathway alone does not induce invasive growth

**Figure 7:**
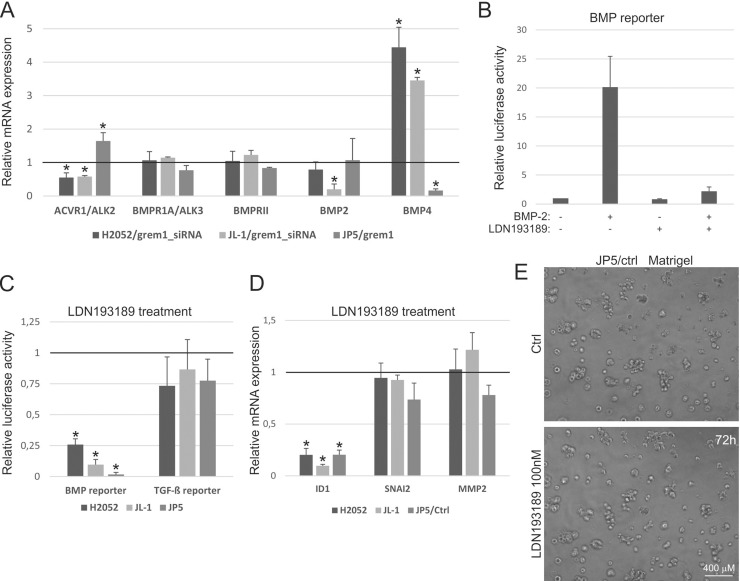
Inhibition of BMP receptor pathway alone does not induce invasive growth (**A**) Expression of BMP-pathway genes in gremlin-1 silenced (grem1_siRNA) H2052 or JL-1 cells and in JP5/grem1 cells. The results are expressed relative to each control (control siRNA transfected or JP5/ctrl), which was set to 1. The error bars represent SD (*n* = 3). ^*^p < 0.05. (**B**) BMP [(Bre)_2_-luc] pathway activity was analyzed in H2052 cells using promoter reporter assays. Promoter activities in BMP-2 (25 ng/ml) and/or LDN193189 (100 nM) treated (24 hours) cells are shown relative to the level in control treated cells, which was set to 1. The error bars represent SD (*n* = 3). ^*^p < 0.05. (**C**) BMP [(Bre)_2_-luc] and TGF-β [(CAGA)_12_-luc] pathway activities were analyzed using promoter reporter assays. Promoter activities in LDN193189 (100 nM) treated cells (3 days) are shown relative to each control, which was set to 1. The error bars represent SD (*n* = 3). ^*^p < 0.05. (**D**) Expression of *ID1*, *SNAI2*, and *MMP2* in LDN193189 (100 nM) treated mesothelioma cells (H2052, JL-1 and JP5/ctrl). The results are expressed relative to each control, which was set to 1. The error bars represent SD (*n* = 3). ^*^p < 0.05. (**E**) Control or LDN193189 (100 nM) treated JP5/ctrl cells were embedded into 3D Matrigel and followed for 72 hours.

Gremlin-1 inhibits mainly the functions of BMP-2 and -4 [5]. We analyzed in our cell systems the expression of these two BMPs as well as the expression of type I (ACVR1/ALK2, BMPR1A/ALK3, BMPR1B/ALK6) and type II (BMPRII) receptors, which mediate BMP pathway signaling. *BMP4* expression was increased in gremlin-1 silenced cells and decreased in JP5/grem1 cells as compared to the controls (Figure 7A). In JL-1 gremlin-1 silenced cells *BMP2* expression was instead decreased. ALK6 receptor expression was low or undetectable in mesothelioma cells (data not shown). ALK2 expression decreased in gremlin-1 silenced cells and increased in JP5/grem1 cells, while ALK3 and BMPRII expression levels did not change (Figure 7A). The small molecule ALK2/ALK3 receptor inhibitor LDN193189 blocked BMP-2 mediated induction of BMP-pathway activity in H2052 cells (Figure 7B). Treatment of mesothelioma cells with LDN193189 for 3 days reduced BMP-pathway activity as expected, but did not induce TGF-β-pathway activity (Figure 7C). In agreement, BMP-pathway target gene *ID1* (inhibitor of DNA binding 1) mRNA was reduced, while *SNAI2* and *MMP2* expression remained unaltered after the inhibitor treatment (Figure 7D). Invasive growth of JP5/ctrl cells into Matrigel (Figure 7E) or collagen (data not shown) matrices was not enhanced by LDN193189 treatment. These data indicate that gremlin-1 has a notable effect on the BMP signaling system in mesothelioma cells, but the inhibition of ALK2/ALK3 mediated BMP signals alone does not induce mesothelioma cell invasive growth.

### JP5/grem1 xenograft tumors are more vascular and show a tendency to send metastasis

**Figure 8:**
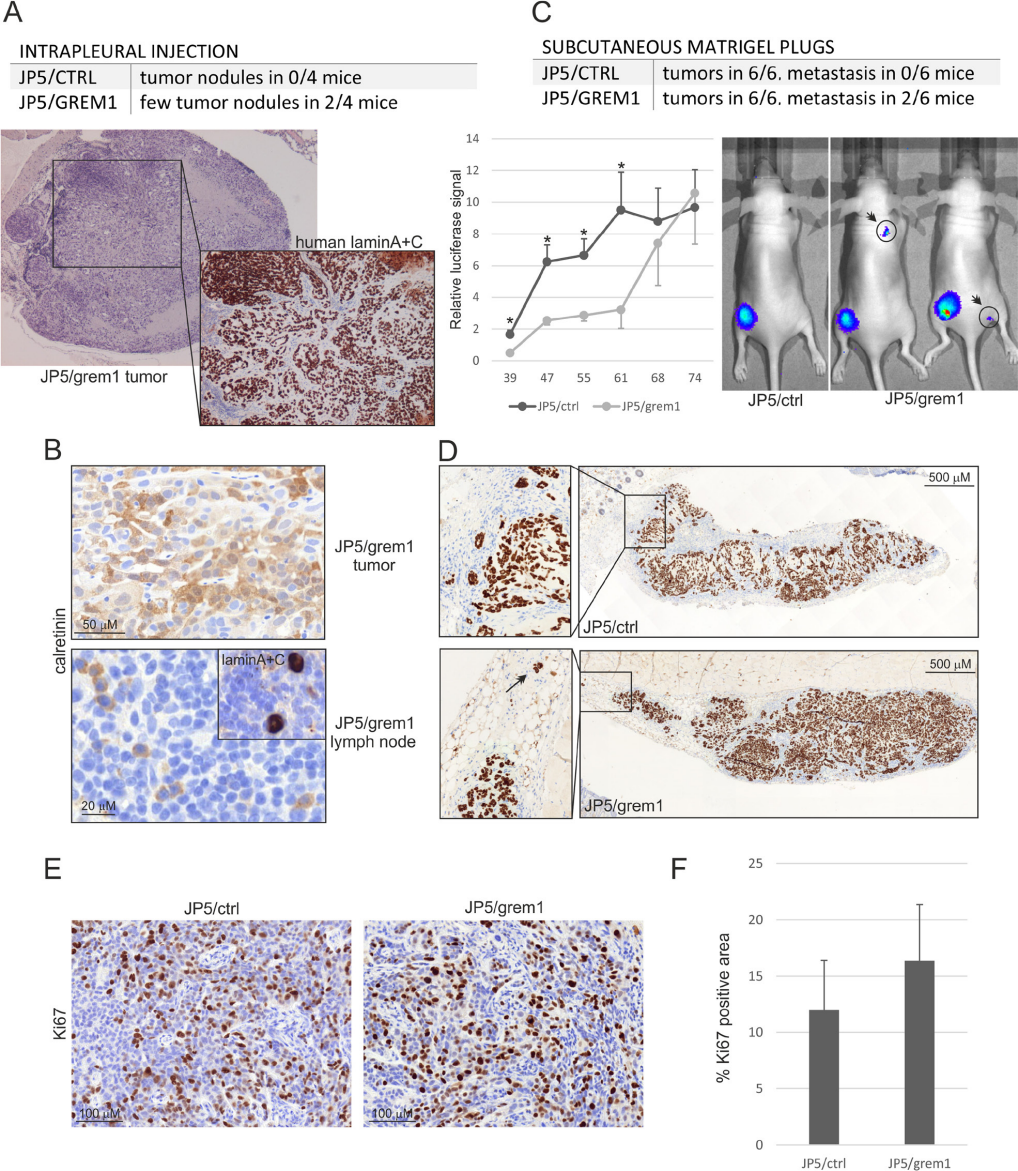
JP5/grem1 xenograft tumors show a tendency to send metastasis (**A**) JP5/ctrl and JP5/grem1 mesothelioma cells transduced to express a luciferase marker gene were injected into the pleural cavity of nude mice and followed for 5 months (*n* = 4). Tumor nodules were analyzed by HE staining and human cells were identified with an antibody specific for human lamin A+C. (**B**) Immunohistochemical staining of JP5/grem1 tumor or an adjacent lymph node with an antibody specific to mesothelial marker calretinin. Inset: staining of the lymph node with lamin A+C antibody. (**C**) JP5/ctrl and JP5/grem1 mesothelioma cells transduced to express a luciferase marker were injected subcutaneously as Matrigel plugs. Tumor growth was followed by luciferase signal measurements for 2.5 months. The error bars represent SEM (*n* = 6). ^*^p < 0.05. Formation of metastasis in JP5/grem1 mice is shown on the right. (**D**) Subcutaneous JP5/ctrl and JP5/grem1 tumors stained with an antibody specific to human lamin A+C. (**E**) Staining of tumors using Ki67 specific antibody. (**F**) The graph shows Ki67 positive staining area (%) in JP5/ctrl and JP5/grem1 tumors. The error bars represent SD (*n* = 6).

A pilot *in vivo* study was performed using orthotopic injection of JP5/ctrl and JP5/grem1 cells into the pleural cavity of nude mice. Tumor initiation and growth was slow, after 5 months follow up no visible tumors or luciferase signal were observed in JP5/ctrl cell injected mice (Figure 8A). In JP5/grem1 cell injected mice formation of a few tumor nodules was observed in 2/4 mice. Histologically the tumors showed epithelioid pattern and calretinin positivity, which is consistent with the original patient tumor type (Figure 8A–8B). In one JP5/grem1 tumor bearing mouse the adjacent lymph node was collected and stained for human calretinin. Isolated calretinin positive cells were detected suggesting that the tumor cells can travel to lymph nodes (Figure 8B). Isolated positive cells were also detected using the human lamin A+C antibody, confirming the human origin of the cells.

To enhance tumor initiation we next injected JP5/ctrl and JP5/grem1 cells subcutaneously as Matrigel plugs into nude mice (*n* = 6, [17]). All mice developed tumors (Figure 8C–8D). In JP5/grem1 cell injected mice tumor development was delayed; however, the tumor growth in this group enhanced and at the time of sacrifice (2.5 months) no difference in luciferase signal was observed. Proliferation marker protein Ki67 staining indicated no significant differences between JP5/ctrl and JP5/grem1 tumors (Figure 8E–8F). In both groups, tumor cells were able to invade into the surrounding tissue as single cells or small colonies (Figure 8D). Although histologically the tumors did not differ notably, there was a tendency to form metastasis in JP5/grem1 tumor bearing mice. In 2/6 JP5/grem1 mice but not in JP5/ctrl mice formation of metastasis was observed (Figure 8C).

**Figure 9:**
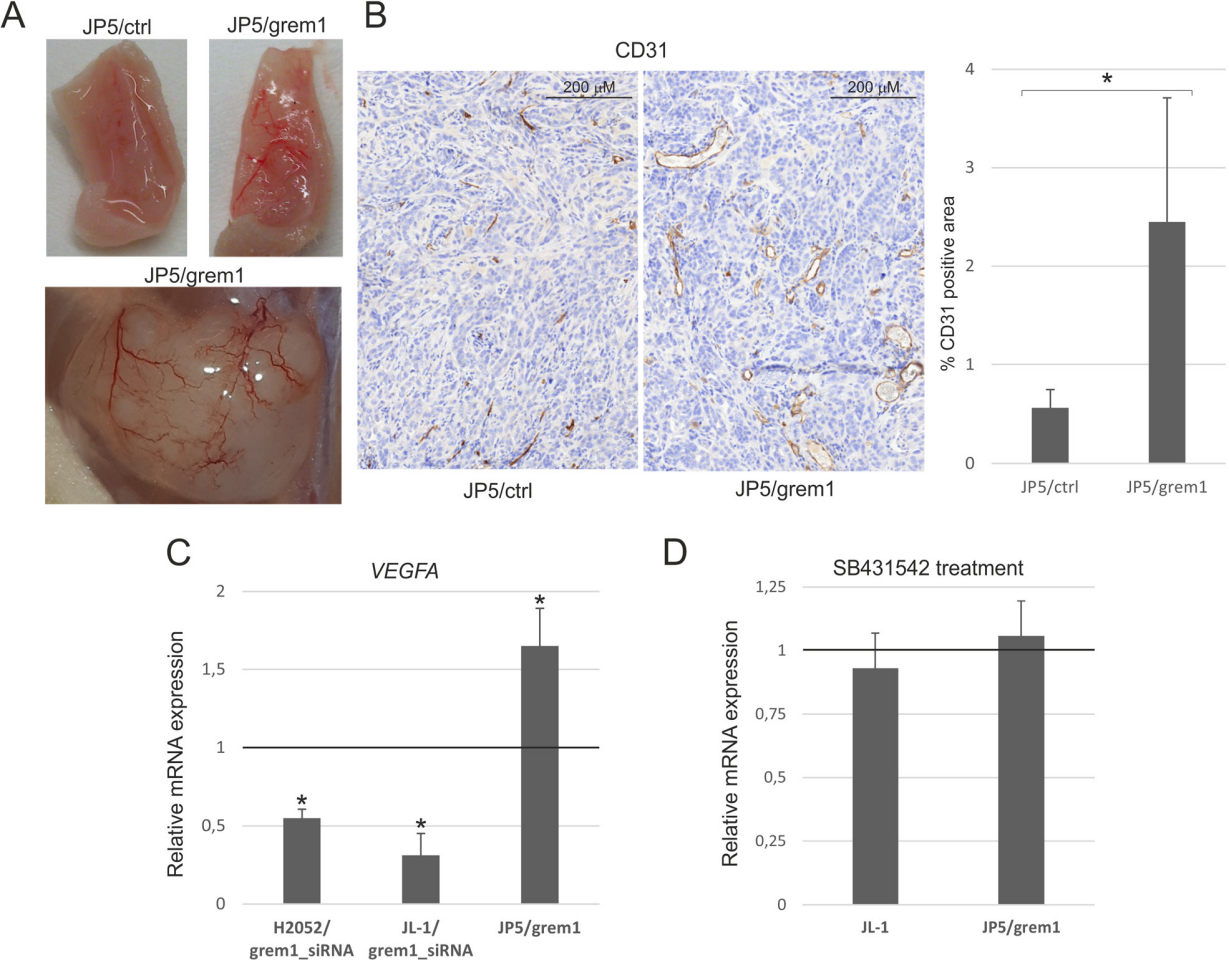
JP5/grem1 tumors are more vascular (**A**) Tumor blood vessels imaged by photography. (**B**) Staining of tumor blood vessels using mouse CD31 specific antibody. The graph shows CD31 positive staining area (%) in JP5/ctrl and JP5/grem1 tumors. The error bars represent SD (*n* = 6). ^*^p < 0.05. (**C**) Expression of VEGFA gene in gremlin-1 silenced (grem1_siRNA) H2052 or JL-1 cells and in JP5/grem1 cells. The results are expressed relative to each control (control siRNA transfected or JP5/ctrl), which was set to 1. The error bars represent SD (*n* = 3). ^*^p < 0.05. (**D**) Expression of *VEGFA* in SB431542 treated mesothelioma cells (JL-1 and JP5/grem1). The results are expressed relative to each control (DMSO treated cells), which was set to 1. The error bars represent SD (*n* = 3). ^*^p < 0.05.

Of note was the high vascularization of JP5/grem1 tumors. The tumors contained visibly more blood vessels (Figure 9A). In agreement, staining of the tumor tissue with CD31 antibodies showed more staining and larger vessels in JP5/grem1 tumors (Figure 9B). Gremlin-1 can signal through the VEGFR2 and it has also been recently linked to the regulation of VEGF expression in pigmentation epithelial cells [8, 18]. Notably, *VEGFA* mRNA expression was decreased in gremlin-1 silenced mesothelioma cells and increased in JP5/grem1 cells as compared to the controls (Figure 9C). Inhibition of TGF-β pathway activity with SB431542 did not alter VEGFA expression in JL-1 and JP5/grem1 cells. Gremlin-1 may thus have angiogenic functions either directly through VEGFR2 or by inducing VEGFA expression. Altogether, these data indicate that by inducing a mesenchymal invasive cell phenotype together with enhanced tumor vascularization, gremlin-1 drives mesothelioma invasion and metastasis.

## DISCUSSION

Recently, gremlin-1 has emerged as a regulator of malignant cell behavior. Gremlin-1 can function as a niche factor modulating cell proliferation as well as regulates plasticity and stemness in cancer cells [7, 19, 20]. In colorectal cancer, gremlin-1 has been localized to cancer invasive fronts in the tumor tissue [21]. In addition to inhibition of BMP-pathway activities, gremlin-1 can function through activation of VEGFR2 signaling and inhibition of MIF [8, 22]. Mesothelioma develops from mesothelial cells lining serosal membranes, most often from the pleura. Asbestos exposure is a causative factor in mesothelioma and we have previously shown that asbestos fibers can increase gremlin-1 expression in lung epithelial cells *in vitro* [23]. Normal mesothelial cells express low levels of gremlin-1, while non-malignant reactive pleural mesothelial cell already show activation of gremlin-1 expression [12]. Mesothelioma cells in tumor tissue display high gremlin-1 immunoreactivity in both epithelioid and sarcomatoid tumor areas. In addition, primary mesothelioma cells isolated from patient pleural fluids show high gremlin-1 expression. Gremlin-1 is targeted into fibrillin-2 containing microfibrils in the tumor microenvironment. Our present data suggest that gremlin-1 functions as an important regulator of the invasive growth behavior of mesothelioma cells *in vitro* and *in vivo*.

In some studies overexpression of gremlin-1 was found to induce proliferation in cultured epithelial or mesenchymal cells [24, 25]. Consistent with this, we observed previously that gremlin-1 silencing in H2052 mesothelioma cells reduces proliferation [12]. However, primary mesothelioma cells tend to lose gremlin-1 expression concomitantly with increased growth rate during passaging. In addition, in overexpression studies we have also observed reduced growth rate in gremlin-1 expressing cells. This may suggest that in cultured mesothelioma cells adapted to proliferate in 2D cultures the balance of gremlin-1 expression affects both proliferation and cell phenotype. Our data clearly suggest that gremlin-1 drives an EMT-phenotype in mesothelioma cells, which is often linked to reduced proliferative activity. This mesenchymal transition of mesothelial/mesothelioma cells was strongly linked to invasive growth in 3D collagen matrix, mimicking somewhat better the tumor tissue microenvironment than 2D cultures. *In vivo* studies also suggested that in gremlin-1 overexpressing cells tumor development was slower, but there was a tendency to send metastasis. Gremlin-1 may thus play a role in mesothelioma cell invasion and dissemination rather than in the induction of high proliferative rate. The EMT transcription factor *SNAI2* was previously shown to co-localize with gremlin-1 in mesothelioma tumor tissue and here we also observed that *SNAI2* expression followed tightly gremlin-1 expression in cultured cells [12]. SNAI2 has been linked to self-renewal capacity of cells as well as to the regulation of EMT processes [26, 27] and is likely an important mediator of gremlin-1 function in mesothelioma. In agreement with our data, a recent study identified gremlin-1 expression in effluent-derived mesothelial cells in peritoneal dialysis patients, which was associated with mesenchymal cell transition and peritoneal dysfunction [28].

EMT is a developmental program that confers a mesenchymal phenotype to epithelial cells. Cancer cells show plasticity and can undergo mesenchymal transitions, characterized by changes in cell surface molecules, ECM proteins, matrix degrading enzymes and growth factor signaling [27]. We observed gremlin-1 mediated alterations in integrin expression, especially the expression of integrin α_v_ subunit (*ITGAV*) followed gremlin-1 levels. This integrin in complex with β_3_ integrin has been previously shown overexpressed in many types of cancers [29] and in mesothelioma it has been linked to cell attachment and invasion [30]. Cilengitide is an inhibitor of α_v_β_3_ and α_v_β_5_ integrins and was found to inhibit mesothelioma invasion in an agarose spot assay in this study. However, there was only a minor effect in invasion into 3D collagen, which is in agreement with our results. As cell culture models we used gremlin-1 silencing in two different mesothelioma cell lines as well as gremlin-1 overexpression in low gremlin-1 expressing cells. Evidence from these models suggest that also *MMP2* and *MMP14* expressions are regulated by gremlin-1. MMPs can break down ECM to allow cell migration as well as modulate the stroma to promote growth and invasion [31]. In agreement, inhibition of MMP activity prevented gremlin-1 induced mesothelioma cell invasion into 3D collagen.

TGF-β is a well-known regulator of EMT and involved in mesothelioma tumor biology. We have previously observed that by blocking BMP-pathway signaling, gremlin-1 can shift the balance towards increased TGF-β-pathway signaling [14]. There are also reports describing direct activation of the Smad-pathway by gremlin-1 [15]. In mesothelioma cells we observed gremlin-1 mediated regulation of the expression of the activin-A subunit (*INHBA*) as well as *TGFB1* and *TGFB2*. Secretion of TGF-β activity and activation of promoter reporter activities were in agreement with the expression studies. We have previously linked activin signaling to mesothelioma invasive growth in 3D Matrigel [16]; however, inhibition of activin activity was not enough to reduced invasive growth in 3D collagen. A significant reduction was achieved when a small molecule inhibitor of TGF-β pathway receptors was used, evidence of the involvement of TGF-β/activin pathways in mesothelioma invasive growth. To get more insight into the importance of TGF-β pathway activity in gremlin-1 induced molecular changes, we analyzed mRNA expression after treatment of cells with the TGF-β receptor inhibitor. Interestingly, the expressions of *MMP2* and *PAI1*, which is a known TGF-β target gene, was downregulated while *SNAI2* and *MMP14* expressions remained unchanged. These data suggest that activation of TGF-β pathway activity is only partially responsible for gremlin-1 mediated processes in mesothelioma cells.

Tumor xenograft studies are important to understand invasive growth of cancer cells *in vivo*. The *in vivo* tumorigenicity and growth of patient-derived mesothelioma xenografts in nude mice can vary a great deal [32, 33]. We observed slow growth and tumor development when using JP5 mesothelioma cells, gremlin-1 tumors taking more time to grow in the early phases of tumor development. Although the number of mice used in the studies was small, a clear tendency for gremlin-1 expressing mesothelioma cells to invade into lymph nodes and to metastasize was identified. In previous studies lymph node metastasis have also been noted in the later stages of xenograft growth in nude mice [32]. Gremlin-1 was also observed to be angiogenic, which is likely to contribute to the ability to form metastases from subcutaneous tumors. Hypoxia has been shown to induce gremlin-1 expression in the context of pulmonary hypertension [34]. In addition, gremlin-1 acts by binding and activating the VEGFR2 in endothelial cells to stimulate angiogenesis [8]. Here, we observed gremlin-1 mediated upregulation of VEGFA expression in mesothelioma cells. Angiogenesis is a prognostic factor in mesothelioma [35] and therapeutic interventions targeting angiogenic growth factor pathways have been tested in clinical trials. Addition of anti-VEGF antibody therapy to standard chemotherapy regimen was shown to offer a survival benefit of 2.7 months in a phase III clinical trial [36], while in another study small molecule inhibitor of VEGF receptors did not offer any clinical benefit [37, 38]. Mesothelioma tumors produce other angiogenic factors including FGFs, TGF-β and gremlin-1 [35] and further clinical studies are needed to assess the usefulness of anti-angiogenic therapies in mesothelioma.

Gremlin-1 is consistently upregulated in mesothelioma [39] and drives a migratory and invasive mesenchymal phenotype. Mesenchymal transition is linked to chemoresistance and cancer prognosis [27]. Targeting gremlin-1 in mesothelioma tumor tissue may present a way to decrease chemoresistance and invasive growth by affecting multiple tumorigenic processes including mesenchymal programming and angiogenesis.

## MATERIALS AND METHODS

### Plasmids and siRNAs

Human gremlin-1 cDNA was amplified by polymerase chain reaction (PCR) using the following primers: 5′-CCGCTCGAGATGAGCCGCACAGCCTA CAC-3′ and 5′-GCTCTAGATTAATCCAAATCGATG GATATGC-3′ with XhoI and XbaI restriction sites, respectively. Gremlin-1 cDNA was then cloned into a lentiviral expression vector pLVX-puro (Clontech) and verified by DNA sequencing. Gremlin-1 is produced from this construct as a native protein without tags. LUC2 firefly luciferase gene (Promega) in pLVX-hygro vector was used as a reporter in *in vivo* studies. Gremlin-1 siRNAs and a negative control siRNA were from Life Technologies.

### Reagents and antibodies

Anti-gremlin-1 antibody was from Origene (TA324077), anti-E-cadherin antibody from BD Biosciences (610182) and anti-β-tubulin antibody from Santa Cruz (sc-9104). Integrin alpha v blocking antibody was from Millipore (MABT207) and used at 10 μg/ml concentration. Antibodies used for immunohistochemistry were anti-human calretinin (Abcam, ab16694), anti-mouse CD31 (Abcam, ab124432), anti-Ki67 (Abcam, ab16667) and anti-human laminA+C nuclear envelope marker (Abcam, ab108595), which was used to identify human tumor cells in mouse tissue. Broad spectrum MMP inhibitors GM6001 and BB2516 were from Calbiochem and used at 10 μM concentration. TGF-βR inhibitors SB431542 and SB505124 were from Sigma-Aldrich and used at 10 μM concentration. BMP receptor inhibitor LDN193189 was from Sigma-Aldrich and used at 100 nM concentration. BMP-2 was from R&D Systems and used at 25 ng/ml concentration. The recombinant fusion protein containing the ectodomain of human ActR2B (sActR2B-Fc) or anti-Müllerian hormone receptor (sAMHR2-Fc) fused to the Fc domain of human IgG1 and recombinant follistatin were produced as described previously [40] and used at 10 μg/ml. Soluble receptors sequester ligands and inhibit their binding to cell surface receptors.

### Cell culture and transfections

Immortalized normal human mesothelial cells (Met5A) and human mesothelioma cell lines H2052, 211H and H28 were from ATCC. JL-1 mesothelioma cell line was from DSMZ [41]. JP primary mesothelioma cells were acquired from pleural effusion samples from patients suffering from malignant mesothelioma as described [12]. Mesothelioma cells were cultured in RPMI-1640 medium (Sigma-Aldrich). 293FT cells (Invitrogen) were cultured in DMEM medium (Sigma-Aldrich) containing 4.5 μg/ml glucose. Culture media were supplemented with 10% heat-inactivated fetal bovine serum (Thermo Fisher Scientific), 1% L-glutamine, penicillin (100 U/ml), and streptomycin (100 μg/ml). Cells were incubated at 37°C in 5% CO2.

For plasmid and siRNA transfections, cells were detached with trypsin and seeded into plates to grow overnight in growth medium without antibiotics. A mixture of plasmid DNA or siRNA with Lipofectamine 2000 transfection reagent (Invitrogen, Life Technologies) was added to the cells according to the manufacturer's protocol. The medium was changed to normal growth medium 6 hours after transfection.

### Lentivirus production and cell transduction

Lentiviruses were produced in 293FT cells using Lipofectamine 2000 (Invitrogen) transfection reagent. Supernatants of 293FT transfectants were collected 48 hours after transfection and filtered using 0.45 μm filter units (Sartorius). JP5 cells were grown to 50–60% confluency and transduced with lentiviral particles in the presence of hexadimethrine bromide (10 μg/ml Polybrene, Sigma-Aldrich). Cells stably expressing firefly luciferase (LUC2) or human gremlin-1 were obtained by selection with hygromycin (200 μg/ml) or puromycine (20 μg/ml), respectively. Cells expressing LUC2 were further transduced with lentiviral particles containing either pLVX-puro empty vector or pLVX-puro carrying human gremlin-1. Western blotting against gremlin-1 was performed to assess the overexpression efficiency.

### RNA isolation and quantitative RT-PCR

Total cellular RNA was isolated using RNeasy Mini kit (Qiagen) and reverse transcribed to cDNA using iScript cDNA synthesis Kit (Bio-Rad). The cDNAs were amplified using TaqMan Assays-on-Demand gene expression products (Applied Biosystems) and CFX96 Real-time PCR detection system (Bio-Rad). The relative gene expression differences were calculated with the comparative ΔΔCT method and the results have been expressed as mRNA expression levels normalized to the levels of a gene with a constant expression (TBP, TATA-binding protein). Pathway-specific PCR array (extracellular matrix and adhesion, SABiosciences) was performed according to manufacturer's instruction.

### 3D growth and invasion assays

Cell invasion assay in 3D Matrigel was performed as described [42]. Cells (2–4 × 10^3^) were plated on 96-well plates pre-coated with 50 μl Matrigel (BD Biosciences, diluted 1:3 in serum-free culture medium), followed by another layer of Matrigel (45 μL) above the cells. Finally, normal cell growth medium containing 10% serum was added on top. Inhibitors were added into Matrigel and growth medium where indicated. The growth patterns and morphologies of the cells were followed and documented by microscopic photography using an AxioVert 200 microscope (Carl Zeiss).

The 3D collagen invasion assay was performed as described [43]. Briefly, cell spheroids were obtained by overlaying cells in 0.5% agarose coated 96-well round bottom plate. Spheroids were then picked up, mixed with type I collagen (Sigma) and dropped on the surface of a culture plate. After collagen polymerization, normal growth medium was added. Growth and sprouting of cells from spheroids was monitored and photographed using AxioVert 200 microscope (Carl Zeiss). The spheroid surface area was determined using FijiImageJ 64 bit software by delineating the surrounding border of cell spheres with cellular protrusion and nodular extension in 20 random images of three to six separate gels. Subsequent quantification of the delineated area was also performed with the same software. Fold change was calculated by comparing the spheroid surface area to the surface area at time point zero, which was set to one. At least three independent experiments were performed.

### Western blotting analysis

Western blot analyses of whole-cell protein lysates (RIPA lysis buffer supplemented with protease inhibitors, Thermo Fisher Scientific) and secreted proteins were performed as described [12]. Protein concentrations were assayed using a BCA protein assay Kit (Pierce, Rockfors, IL, USA). Equal amounts of protein were separated by SDS-PAGE using 4–20% gradient Tris-glycine gels (Bio-Rad) and transferred to nitrocellulose membranes (Bio-Rad). After incubation with primary and secondary antibodies, the proteins were detected using Odyssey imaging (Li-Cor Biosciences). Relative band densities were quantified using Image Studio Lite Ver 5.2 software (Li-Cor Biosciences).

### Gelatin zymography

Concentrated cell conditioned medium (10×, Microcon concentrators, Millipore) was analyzed by gelatin zymography using precast 10% SDS-PAGE gels containing gelatin (Bio-Rad). The amount of media loaded into the gel was adjusted according to cell number, which was counted after the collection of the media. Conditioned medium from HT-1080 cells treated with 4 nM PMA was used as a control. After electrophoresis the gels were processed and stained with Coomassie Brilliant Blue (Bio-Rad) as described [44]. Scanning of gels was performed using Epson Perfection 4490 Photo Scanner, and quantification using FijiImageJ 64 bit program.

### Staining of F-actin

Cells were cultured on glass coverslips, fixed with 3.5% (v/v) paraformaldehyde in PBS and permeabilized with 0.1% Triton X-100 in PBS. Filamentous actin was then stained with TRITC-conjugated phalloidin (Sigma-Aldrich). The coverslips were mounted in Vectashield containing DAPI (Vector Laboratories). Fluorescent images were visualized and recorded using Zeiss Axio Imager.Z2 microscope (Carl Zeiss).

### Cell adhesion assay

Cells were suspended in culture medium and plated into plasma fibronectin (pFN, 10 μg/mL in PBS, Sigma-Aldrich) or collagen I (10 μg/mL in PBS, BD Bioscience) coated 96-well plates. Bovine serum albumin was used as a control. Cells were allowed to attach for 60 min at 37°C, after which cell morphologies were documented with photography. Finally, the percentage of attached and spread cells was calculated. At least 100 cells in three randomly selected fields were analyzed.

### Cell migration assays

The migratory capacity of JP5 cells was analyzed using Incucyte ZOOM 2013A kinetic live cell imaging system (Essen Bioscience). The cells were plated on 96-well plates and the next day a wound was applied using a wound maker (Essen Bioscience). The media was replaced with serum-free media. Two distinct pictures were taken of each well every 2 hours for 24 hours. Data were analyzed with Incucyte ZOOM 2013A software and the relative wound density was used as a measure of the wound closure.

Transwell cell migration/invasion assays were performed as described previously [42]. The inserts (8 μm pores, Corning Costar) were coated on the outer surface with collagen-I (45 μg/ml, BD Bioscience) and the lower chamber was filled with serum-free medium. Alternatively, the inserts were used without coating and the lower chamber was filled with medium containing 1% serum as chemoattractant. Cells (1–2 × 10^4^) in serum-free medium were then added to the upper chamber. After an 18-hour incubation the insert filters were fixed in 3.5% paraformaldehyde and stained with 0.5% crystal violet (in 20% methanol). Cells on the upper side of the filter were scraped off, and migrated cells on the lower side were photographed and counted. Experiments were repeated at least three.

### TGF-β/BMP luciferase reporter assays

Cells to be transfected were seeded in 96-well plates. The cells were co-transfected with promoter constructs (CAGA)_12_-luc (Smad3 responsive) or (Bre)_2_-luc (Smad1/5 responsive), kindly provided by Dr. Peter ten Dijke (Leiden University Medical Center, the Netherlands), together with pRL-TK (Renilla luciferase control, Promega) plasmid using Fugene HD transfection reagent (Roche). After a three-day incubation, the cells were lysed and subjected to luciferase activity measurement by Dual Luciferase Reporter Assay (Promoga) and DCR-1 luminometer (MGM Instruments Digene Diagnostics Inc.). The luciferase activities were normalized to constitutively expressed Renilla luciferase activities, and the results are expressed relative to control.

### TGF-β activity assay

Mink lung epithelial cells stably transfected with a fragment of the human plasminogen activator inhibitor-1 promoter fused to the firefly luciferase reporter gene (TMLC) were kindly provided by Dr. Daniel Rifkin (NYU School of Medicine, New York). The cells respond to TGF-β with luciferase activity and were used to assess the amount of active and total TGF-β present in the cell conditioned media. For measurement of total TGF-β activity, heat treatment (80°C, 5 min) was used to activate latent forms of TGF-β. Analysis of TGF-β in standards and medium samples was performed as described [45]. The results have been presented as relative values of TGF-β activity.

### Orthotopic pleural xenografts and subcutaneous xenografts

All experiments involving animals were approved by the Provincial State Office of Southern Finland (ESAVI/2083/04.10.07/2015) and carried out in accordance with institutional guidelines, which fulfill the requirements defined in regulations of the Finnish Act on the Protection of Animals used for Scientific or Educational Purposes (497/2013) and were performed according to the 3R. Female 6-week-old Balb/c nu/nu mice (Scanbur) were used. Orthotopic pleural xenograft model was applied to mimic the human mesothelioma microenvironment. JP5 cells (1 × 10^6^) expressing firefly luciferase together with human gremlin-1 (JP5/grem1) or control vector (JP5/ctrl) were directly injected into the pleural cavity through the intercostal space. Subcutaneous xenografts were performed by injecting JP5 cells (1 × 10^6^) mixed with Matrigel Matrix (BD Biosciences, final concentration 7 mg/ml) into the flank region of mice. The procedures were performed under isoflurane anesthesia and the mice were observed until recovery. Successful injection of tumor cells as well as tumor growth was followed by bioluminescence imaging. D-Luciferin (Regis Technologies) in PBS was injected intraperitoneally and allowed to circulate for 10 min. Emitted photons were monitored with IVIS Kinetic whole animal imaging system (Perkin-Elmer). The weight of mice was monitored regularly throughout the experiments. Tumor tissue of sacrificed mice was collected, fixed in 4% paraformaldehyde, dehydrated and embedded in paraffin.

### Immunohistochemistry

Paraffin-embedded tissue samples were processed and stained using the Leica BOND-MAX fully automated staining system (Leica Bond Polymer Refine Detection-kit and Bond Epitope Retrieval Solution 1, 20 mins). The primary antibody incubation was 60 min. Negative control sections were incubated with rabbit isotype control (Invitrogen). The slides were scanned digitally (Pannoramic FLASH II, 3DHistech) and analyzed using CaseViewer program and HistoQuant quantification module (3DHistech).

### Statistical analyses

All comparisons were made using nonparametric tests with SPSS version 24 software (IBM). Two-group comparisons were made using Mann-Whitney *U*-test. *P* values below 0.05 were considered statistically significant.

## SUPPLEMENTARY MATERIALS FIGURES



## Abbreviations

TGF-β: transforming growth factor-β
3D: three dimensional
BMP: bone morphogenetic protein
VEGFR2: vascular endothelial growth factor receptor 2
MIF: migration inhibitory factor
EMT: epithelial-to-mesenchymal transition
MMP: matrix metalloproteinase
ALK: activin receptor-like kinase
