# Supplementary material for: Gremlin-1 is a key regulator of the invasive cell phenotype in mesothelioma
Source: Oncotarget. 2017 Oct 6;8(58):98280–97. doi: 10.18632/oncotarget.21550 (PMC5716729; doi:10.18632/oncotarget.21550)
Supplement: Supplementary file 1 [file oncotarget-08-98280-s001.pdf]

Gremlin-1 is a key regulator of the invasive cell phenotype in mesothelioma

SUPPLEMENTARY MATERIALS

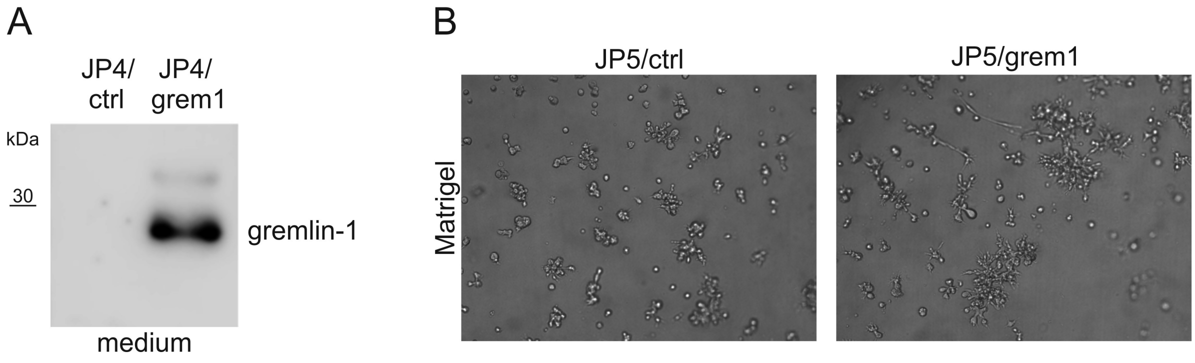

Supplementary Figure 1: (**A**) Western blotting of gremlin-1 from JP4 conditioned media (normalized according to cell number). (**B**) JP5/ctrl and JP5/grem1 mesothelioma cells were embedded into 3D Matrigel and followed for 72 hours.

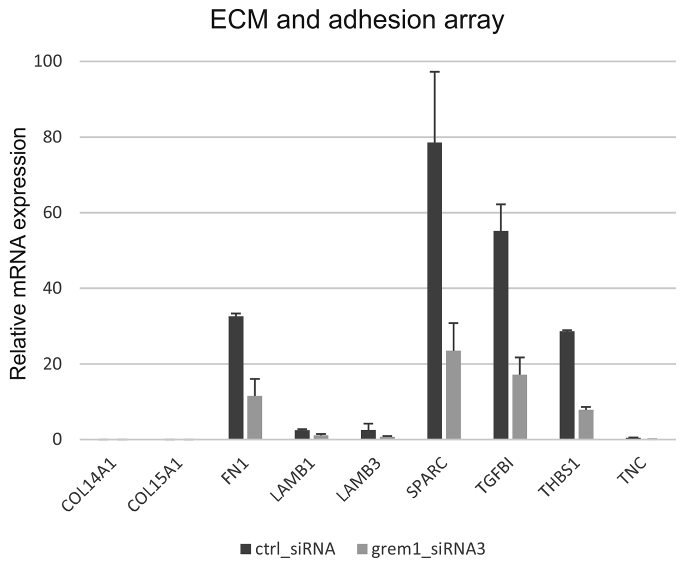

Supplementary Figure 2: Extracellular matrix and adhesion molecule PCR array was used to analyze gene expression in control siRNA (ctrl_siRNA) or gremlin-1 siRNA (grem1_siRNA3) transfected H2052 cells 3 days after transfection (*n* = 2).

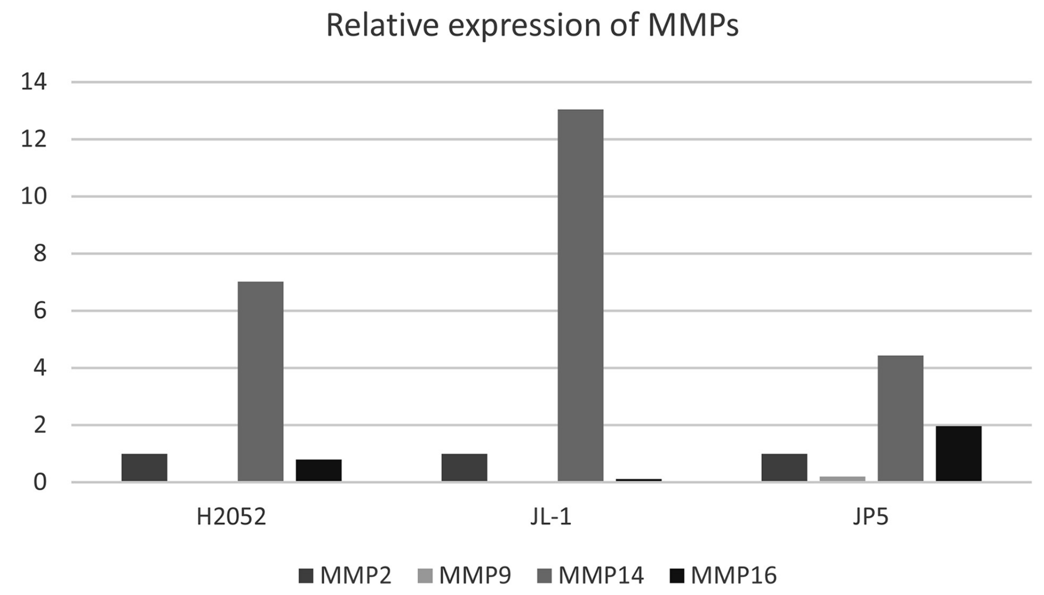

Supplementary Figure 3: Relative expression levels of MMP genes in mesothelioma cells. The results are expressed relative to MMP2 level in each cell line, which was set to 1. A representative experiment is shown.
